# Oxidative stress and senescence in social insects: a significant but inconsistent link?

**DOI:** 10.1098/rstb.2019.0732

**Published:** 2021-04-26

**Authors:** Boris H. Kramer, Volker Nehring, Anja Buttstedt, Jürgen Heinze, Judith Korb, Romain Libbrecht, Karen Meusemann, Robert J. Paxton, Alice Séguret, Florentine Schaub, Abel Bernadou

**Affiliations:** ^1^ Faculty of Science and Engineering, Theoretical Research in Evolutionary Life Sciences, RUG, 9747 AG Groningen, The Netherlands; ^2^ Department of Evolutionary Biology and Ecology, Institute of Biology I (Zoology), University of Freiburg, Hauptstraße 1, 79104 Freiburg (Brsg.), Germany; ^3^ Institute for Biology - Molecular Ecology, Martin-Luther-University Halle-Saale, Hoher Weg 4, 06099 Halle, Germany; ^4^ Zoology, Department of Evolutionary Biology, University of Regensburg, Universitätsstraße 31, 93053 Regensburg, Germany; ^5^ Institute of Organismic and Molecular Evolution (IOME), Johannes Gutenberg University Mainz, Hanns-Dieter-Hüsch-Weg 15, 55128 Mainz, Germany; ^6^ Institute for Biology - General Zoology, Martin Luther University Halle-Wittenberg, Hoher Weg 8, 06120 Halle, Germany

**Keywords:** social insects, ageing, longevity, protein oxidation, antioxidant genes, transcriptomes

## Abstract

The life-prolonging effects of antioxidants have long entered popular culture, but the scientific community still debates whether free radicals and the resulting oxidative stress negatively affect longevity. Social insects are intriguing models for analysing the relationship between oxidative stress and senescence because life histories differ vastly between long-lived reproductives and the genetically similar but short-lived workers. Here, we present the results of an experiment on the accumulation of oxidative damage to proteins, and a comparative analysis of the expression of 20 selected genes commonly involved in managing oxidative damage, across four species of social insects: a termite, two bees and an ant. Although the source of analysed tissue varied across the four species, our results suggest that oxidative stress is a significant factor in senescence and that its manifestation and antioxidant defenses differ among species, making it difficult to find general patterns. More detailed and controlled investigations on why responses to oxidative stress may differ across social species may lead to a better understanding of the relations between oxidative stress, antioxidants, social life history and senescence.

This article is part of the theme issue ‘Ageing and sociality: why, when and how does sociality change ageing patterns?'

## 1. Introduction

Social insects are excellent model organisms to investigate the evolution of senescence. Owing to division of labour, genetically similar individuals can express two or more distinct phenotypes that differ vastly in their lifespans. Reproductive individuals, even though they carry the physiological costs of reproduction, can have extraordinary lifespans of up to 30 years, while non-reproductive workers die much younger [1–3]. These findings challenge life-history theory, which assumes that limited resources lead to trade-offs between somatic and reproductive functions.

### (a) Oxidative stress

Oxidative stress reflects an imbalance between the systemic manifestation of reactive oxygen species (ROS) and the organism's ability to neutralize either the reactive molecules or the damage that they cause; the latter represents a proximate cause for senescence. Metabolically demanding activities such as reproduction should lead to increased production of ROS. As a side-product of mitochondrial metabolism, ROS are released that can cause oxidative stress by damaging important biomolecules such as proteins, lipids or nucleic acids [4], but can also have an important function in homeostasis and cellular signalling [5].

Here our focus is on social insects, among which experimentally induced ROS increase senescence of social bees and ants [6–9]. One effect of ROS is the oxidation of amino acids in proteins by introducing carbonyl groups, which often leads to a loss of catalytic activity and simultaneously marks the protein for proteolytic degradation (reviewed in [10] and [11]). Carbonyl content increases with age in a variety of solitary species, especially in the last third of the lifespan (reviewed in [10]). If this damage is not prevented or repaired, it can interfere with normal cellular function. Thus, the detection of protein carbonylation is widely used as a measure of senescence, i.e. a functional decline of the organism with increasing age [12,13].

Senescence owing to ROS can be prevented. Individuals can either avoid producing ROS, or they can quickly neutralize occurring ROS using antioxidants, or they can repair the damage caused by ROS. Some antioxidants can be produced by the individuals themselves, such as superoxide dismutase (*Sod*), catalases and peroxidases, that act directly on ROS to prevent oxidative damage [13–16]. Antioxidant processes are of particular interest in social insects as they might explain how reproductive individuals maintain extremely high fertilities associated with higher metabolic activity across their extended lifespans without showing signs of senescence, including oxidative stress [17,18].

However, evidence for enzymatic antioxidants as a major predictor of lifespan is mixed. Increased expression of antioxidant-encoding genes occurs in young queens and old workers, but not in old queens in *Apis mellifera* honeybees and *Lasius niger* ants [13,19,20], and the activity of antioxidant enzymes is not necessarily higher in long-lived castes [8,19,20]. In *Reticulitermes* termites, in which reproductives outlive workers and accumulate less damage to DNA, proteins, and lipids, *catalase* and *peroxiredoxin* gene expression is higher in queens, and so is catalase activity [16,21]. While the expression of superoxide dismutase genes does not differ between workers and reproductives, *Sod* activity is higher in reproductives [22].

Vitellogenin (Vg) production, on the other hand, is consistently linked to lifespan. These proteins are synthesized in the fat body and circulate in the haemolymph before they are taken up into oocytes where they serve as nutrients for the developing embryo [23]. As Vg is more abundant in long-lived queens than in short-lived workers, and as it also correlates with lifespan among workers, it has been suggested that Vg protects honeybees from oxidative stress [6,7,24,25]. When oxidative stress is exogenously induced in honeybee workers, Vg becomes significantly more oxidized than other haemolymph proteins, suggesting an antioxidant function for Vg [6]. When honeybee workers transition to foraging and hence begin to fly, circulating Vg titres drop, damaged proteins accumulate in some tissues [26,27] and workers become more susceptible to oxidative stress and other stressors [28]. It has been argued that such damage is unique to flying insects. This may be because the flight is metabolically much more demanding than foraging on the ground, as in ant or termite workers, and might thus temporarily produce more ROS than the organism can withstand [14]. However, so far there is no evidence that proteins are specifically damaged during foraging flights [29,30].

Generally, despite a vast body of research on oxidative stress, it is not known whether oxidative damage consistently accumulates with age and whether similar genes/mechanisms are involved in protection against stress across social insects [14,31,32]. This is not necessarily surprising because both the effects of ROS and the mechanisms to reduce their production or neutralization are complex. Oxidative damage may not be found because individuals either produce fewer ROS, neutralize them, or repair the damage. Oxidative damage may be found but have no effect on function or lifespan when individuals restrict it to tissues that are not critical for personal fitness (e.g. optic lobes or flight muscles of ant queens; [14,33]). Further, biochemical oxidative stress might not necessarily translate into biological oxidative stress if, for example, the signalling activity of ROS stimulates mechanisms that protect the organism [34].

Here, we aimed to close the knowledge gap (i) by quantifying protein oxidation levels as a direct measure of oxidative stress and (ii) by concurrently analysing changes in the expression of selected genes involved in the enzymatic antioxidant system of workers (non-reproductive individuals) and reproductive individuals across the phylogeny of social insects: one termite species (*Cryptotermes secundus,* Blattodea, Isoptera), two bee species (*Apis mellifera, Euglossa viridissima*, Hymenoptera, Apoidea) and one ant species (*Platythyrea punctata,* Hymenoptera, Formicidae) (more details in table 1, electronic supplementary material, Methods S1). Specifically, we tested the hypothesis that the costs of colony maintenance may in part lie in physically hard work, leading to elevated metabolism in workers that take over many tasks associated with colonial life (e.g. brood rearing, foraging, nest maintenance). As a result, we predicted that oxidative damage should accumulate with age in workers, while reproductives within colonies should be shielded from this effect. We predicted that the expression of genes involved in oxidative stress management should reflect this pattern. Our results led us to hypothesize that antioxidative processes may vary even across closely related social insect species. This might explain why very few genes have yet been clearly identified as playing a conserved role in explaining species-specific senescence in social insects. If true, we predict that the genes involved in the antioxidant system and their regulation in social insects are species-specific rather than generic to many or all species.

**Table 1.** Overview of the samples used for the measurement of protein oxidation and the expression of antioxidant genes.

species	social complexity/lifespan	samples (N) protein oxidation	samples (N) antioxidant genes	tissue protein oxidation/antioxidant genes
*Cryptotermes secundus* (Hill, 1925) (Kalotermitidae, Isoptera, Blattodea)	low: ‘Cooperative breeders' colony size: 200–400 individuals; totipotent workers; lifespan: queens: up to 13 years workers: at least 4 years	young (7)/old (7) queens young (7)/old (7) kings young (7)/old (7) workers	young (2)/old (2) queens young (2)/old (2) kings young (2)/old (2) workers	whole body (without gut)
*Euglossa viridissima* Friese, 1899 (Apidae, Apoidea, Hymenoptera)	low: ‘Facultative eusocial’ colony size: 1–5 individuals; totipotent workers; lifespan: queens: min 2–6 months workers: 2–6 weeks	solitary nest: young (4)/old (5) queens social nest: young (4)/old (7) queens young (5)/old (3) workers	young (4)/old (6) queens young (4)/old (3) workers	thorax/abdomen
*Apis mellifera* Linneaus, 1758 (Apidae, Apoidea, Hymenoptera)	high ‘Superorganism’ colony size: several thousand individuals; functionally sterile workers; lifespan: queens: 1–5 years workers: 4–6 weeks	young (12)/old (9) workers	—	thorax
*Apis mellifera capensis* Eschscholtz, 1822 (Apidae, Apoidea, Hymenoptera)	high ‘Superorganism’ colony size: several thousand individuals; functionally sterile workers, though functionally reproductive workers can develop as pseudoqueens; lifespan: queens: 1–5 years workers: 4–6 weeks	—	early stage^a^ (2)/late stage^b^ (2) pseudoqueens early stage^a^ (2)/late stage^b^ (2) workers	fat body
*Platythyrea punctata* Smith, 1858 (Formicidae, Hymenoptera)	low: ‘Clonal ant’ colony size: 30–80 individuals; totipotent workers; lifespan: reproductive worker: > 400–500 days non-reproductive workers: approximately 200 days	young (9)/old (9) subordinates	young (5)/old (5) dominants – head and abdomen young (5)/old (5) subordinates – head and abdomen	head + thorax/head, abdomen

^a^early stage: day 3, day 4.

^b^late stage: day 7, day 8.

## 2. Material and methods

We quantified the carbonylation of proteins, typically caused by oxidative stress, in young and old workers of four insect species: one termite, two bee species and one ant (table 1). In addition, we measured carbonylation in young and old reproductives in the termite *Cryptotermes secundus* (kings and queens) as well as in solitary and social reproductives of a facultative social bee (*Euglossa viridissima*). We furthermore studied the expression of genes that have been found to be involved in enzymatic antioxidant defence in other insect model organisms [13,35] in young and old non-reproductives as well as reproductives of all four species. For gene expression in honeybees (*Apis mellifera*), we used workers and pseudoqueens of the African subspecies *capensis* while for the ant *Platythyrea punctata* we used workers (subordinates) and dominants (reproductively active workers). The use of worker-like reproductives, where possible, allowed us to examine the role of reproduction *per se* in modulating gene expression with age. Table 1 gives an overview of the species, castes, ages, number of samples and tissues used for the quantification of protein oxidation and antioxidant gene expression.

### (a) Species studied

The termite *Cryptotermes secundus* (Kalotermitidae) lives in dead sound wood that serves as food and shelter. Colonies have low social complexity with small colonies (maximum 200–300 individuals) and totipotent workers [36]. Workers are immature stages that develop into winged sexuals that disperse (via several nymphal instars) or into neotenic replacement sexuals when the natal reproductives die (via a single moult). Only few workers develop into sterile soldiers (2–5 individuals per colony).

*Euglossa viridissima* (Apoidea) is a facultative eusocial bee species, in which all nests are initiated by a solitary foundress female. Once the first brood has emerged, one or several females from this first brood can remain in the nest and help the foundress female care for a second brood, thus initiating the shift to a social nest structure, in which the foundress / mother is dominant (queen) and one or more daughters are subordinate workers [37,38]. Females of this species are totipotent; a subordinate female can take over the role of the dominant upon the mother's death or departure from the nest [39].

By contrast, the honeybee *A. mellifera* (Apoidea) is obligately eusocial. Honeybee colonies usually consist of several thousand sterile female workers and a single highly fecund queen. Workers normally show distinct age polyethism, with young bees feeding the developing larvae in the hive (nurse bees) and older bees flying out to collect nectar and pollen (forager bees) [40]. The age-defined workers used to determine protein oxidation have been shown to follow this normal age polyethism [41]. *A. mellifera capensis* workers deviate from this normal age polyethism in that they can develop into highly fecund pseudoqueens that produce genetically identical offspring by automixis [42,43].

In the clonal ant species *Platythyrea punctata* (Formicidae)*,* colonies are small and workers can produce clonally identical female offspring from unfertilized eggs by thelytokous parthenogenesis [44]. Despite the absence of morphological or genetic differences among totipotent nest-mates, colonies are characterized by a reproductive division of labour between one (occasionally several) reproductive dominant workers (hereafter, dominants) and the majority of subordinate, non-reproducing workers (hereafter, subordinates) [45].

Information on lifespans is provided in table 1. Full sample information about stock colonies, sample collection, processing and the grouping decisions (young/old) is provided in the electronic supplementary material, Methods (electronic supplementary material, Methods S1 and [35]).

### (b) Determination of protein oxidation

To investigate protein oxidation, we used two methods: (i) a spectrophotometric 2,4-dinitrophenylhydrazine (DNPH) assay for the quantification of carbonyl groups in oxidized proteins and (ii) an anti-DNP Western Blot to detect the presence of specific carbonylated proteins.

Samples (for tissues table 1) were homogenized in lysis buffer, centrifuged and the supernatant stored at −20°C until further processing. Protein concentrations were measured according to Bradford [46] adapted to a microplate reader (electronic supplementary material, Methods S2.1). To determine the proteins damaged by oxidative stress, carbonyl content was quantified with a DNPH assay [47] that was adapted to small sample volumes. Standard curves were created by combining changing proportions (0–100%) of oxidized and reduced BSA ([48]; electronic supplementary material, Methods S2.2).

For the Western Blot analysis, extracted proteins were incubated with DNPH in HCl (derivatization sample) or only with HCl (negative control). After 1 h, Tris, glycerol and SDS were added to stop the reaction and to prepare the samples for SDS polyacrylamide (PA) gel electrophoresis (GE). Electrophoretic separation [49] was performed in 12% SDS PA gels at 175 V. The Unstained Protein Standard Broad Range (10–200 kDa) was used as a protein molecular weight marker. Western blots [50] were incubated with anti-DNP primary antibody, HRP goat anti-rabbit lgG secondary antibody and developed with 3,3,5,5 tetramethylbenzidine (electronic supplementary material, Methods S2.3).

To compare the results of the DNPH assay between species, reproductive status and age, we used a non-parametric multilevel Bayesian modelling approach (brms package; [51]) owing to the limited sample sizes and the non-normal distribution of the data. The model used log-transformed DNPH measures as dependent variable and species (four levels), reproductive status (two levels) and age (two levels) as fixed effect factors. To account for pseudo-replication (several individuals belonging to the same colony), we included colony identity as a random effect in the model. To improve the estimates of group means, the residual standard deviation was modelled using the factor species as a predictor variable. Model convergence was inspected by checking Rhat values [51] and ‘caterpillar plots' (bayesplot package; [52]). Owing to the structure of the model and grouping of the data by species, reproductive status and age, we obtained parameters for each group (e.g. *A. mellifera* worker young) from the model that were then used for nonlinear hypothesis testing (brms package; [51]) to compare all population-level effects or combinations of those against each other. We report the evidence ratios (ER, how likely is the tested hypothesis over its alternative) and the posterior probabilities (PP) of direct pairwise comparisons (brms package; [51]). We considered an ER of greater than 6 (PP greater than 0.85) as a trend (see bold font in table 3), while we considered an ER of greater than 22 (PP = 0.95) as significant (*), as recommended [51]. All analyses were performed and graphs generated with the statistical software R 1.2.1335 [53] (Detailed methods and the R script is available in electronic supplementary material, Methods S6).

### (c) Expression of antioxidant genes

To investigate changes in antioxidant gene expression of young and old reproductive or non-reproductive individuals of *C. secundus*, *A. mellifera capensis*, *E. viridissima* and *P. punctata*, we analysed the expression patterns of genes known to be major components of the enzymatic antioxidant system [13,35]. Genes known to have indirect antioxidant effects such as Vg were excluded [6,13].

Following Corona and Robinson [13], we obtained all gene sequences from the latest *Drosophila melanogaster* genome version (release 6.34) available from FlyBase (FB2020_03, June 2020), from *Anopheles gambiae* as present in the *NCBI* database (consulted in July 2020), and from the *Apis mellifera* official gene set v. 1.0 [54]. We applied a two-step forward and reciprocal search strategy to identify the respective sequences in our four studied species and to assign them unambiguously to the correct genes.

(i) For the genes with more than one sequence available, we generated multiple sequence alignments from the protein coding sequences and then built profile hidden Markov models (pHMMs), which can be considered as a statistical representation of the multiple sequence alignments. The pHMMs were used to identify candidate sequences in the genomes of *C. secundus*, *A. mellifera*, *E. viridissima* and the *de novo* assembled transcriptome of *P. punctata* converted into protein level. In every case, we always kept the best resulting candidate hit. For genes with only one sequence available, we used BlastP [55], implemented in the BLAST suite v.2.9+ [56] on a local basis to search against the genomes or the translated transcriptome assembly. We only considered the best hit as candidate sequences for the reciprocal search.
(ii) To unambiguously assign the candidate sequences to the genes of interest, we used a reciprocal BlastP search against the genome of *Drosophila melanogaster* r. 6.34 and *Apis mellifera* v. 1.0 on the protein level. We considered a sequence as correctly assigned to a gene if the best reciprocal hit criterion was fulfilled (i.e. the best hit was similar to the sequence that had been used for the forward search). From the approximately 60 genes described as major components of the enzymatic antioxidant system [13], we generated a final list of 20 genes that were (i) unambiguously assigned and (ii) present in all the four species studied (electronic supplementary material, Methods S3, table S3).

To investigate expression patterns of these 20 genes in relation to age in the four species of interest, we analysed published RNAseq data for *E. viridissima, A. mellifera capensis* and *C. secundus,* and generated our own RNAseq data for *P. punctata.* For full details on the RNAseq data used in this study, see electronic supplementary material, Methods S3. For each species, we normalized the read counts for all expressed genes using the counts() command of the DESeq2 R package [57], and extracted the normalized counts for the 20 candidate genes.

To determine whether or not the four study species showed different expression patterns of the 20 candidate genes, we performed a principal component analyses (PCA) and a permutational multivariate analysis of variance across all species (PERMANOVA—function ‘adonis', vegan package; [58,59]); to do so, the normalized counts for the 20 candidate genes were entered in the PERMANOVA test as dependent variables and species (four levels), reproductive status (two levels: non-reproductive (worker) and reproductive (king & queen)), age (two levels: young and old) and their interactions as independent variables. For this analysis, we included samples from two different tissues in *P. punctata;* however, using tissues separately did not change the results (data not shown). To analyse whether or not individuals of one species of different reproductive status or age differed in the expression pattern of the 20 candidate genes, we performed a PERMANOVA for each species separately. The normalized counts for the 20 candidate genes were entered in the PERMANOVA test as dependent variables and reproductive status (two levels: worker and reproductive), age (two levels: young and old) and an interaction term between these two factors, as independent variables. Since we had kings in addition to queens and workers in the termites, we ran a second PERMANOVA for this species with three levels for the factor reproductive status. All PERMANOVA tests were based on a Euclidean distance matrix and performed on 9999 permutations. We illustrated the results of the PERMANOVA tests using a PCA with the R functions ‘prcomp' and ‘ggbiplot' on each species separately. We used the ‘res.PCA' function to test the significance of the correlation between each gene's expression and the first two PC axes.

We then focused on the PCs that separated individuals either by reproductive status or by age, and identified genes with loadings deviating from zero by more than 0.8. For these genes, we independently tested whether the raw read counts differed between castes or age groups using a non-parametric U-test (table 2, electronic supplementary material, table S5). We were particularly interested in genes that showed similar patterns in all four species.

**Table 2.** Genes that contributed positively or negatively (correlation coefficient threshold greater than 0.8) to the principal component (PC) axes that significantly separated castes and/or ages. Q, queen; W, worker; PQ, pseudoqueen.

species	gene	PC1 (caste)		Wilcoxon rank sum test	
		correlation	*p*	*p*	direction
*C. secundus*	*Cat*	0.963	0	0.028	Q < W
	*CG31028*	0.804	0.016	0.028	Q < W
	*GstS3*	−0.806	0.016	0.028	Q > W
	*GstZ2*	−0.926	0.001	0.028	Q > W
	*MsrA*	0.859	0.006	0.028	Q < W
	*sepia*	0.896	0.003	0.028	Q < W
	*Sod1*	0.924	0.001	0.028	Q < W
*A. mellifera capensis*	*CG6523*	0.847	0.008	0.342	PQ < W
	*CG8993*	0.871	0.005	0.485	PQ < W
	*GstT3*	0.92	0.001	0.200	PQ < W
	*Mgstl*	0.824	0.012	0.685	PQ < W
	*Prx3*	0.879	0.004	0.057	PQ < W
	*Sod1*	0.838	0.009	0.028	PQ < W
*E. viridissima*	*Mgstl*	−0.962	0	0.003	Q < W
	*Sod1*	−0.891	0	<0.001	Q < W
	*Txl*	−0.901	0	0.004	Q < W
**species**	**gene**	**PC2 (age)**		***p***	**direction**
*E. viridissima*	*Prx3*	−0.835	0	0.138	old > young
species	gene	PC1 (age)		*p*	direction
*P. punctata*	*Prx3*	−0.848	0	0.014	old > young
	*Sod2*	−0.900	0	0.023	old > young

## 3. Results

### (a) Protein oxidation

**Figure 1.**
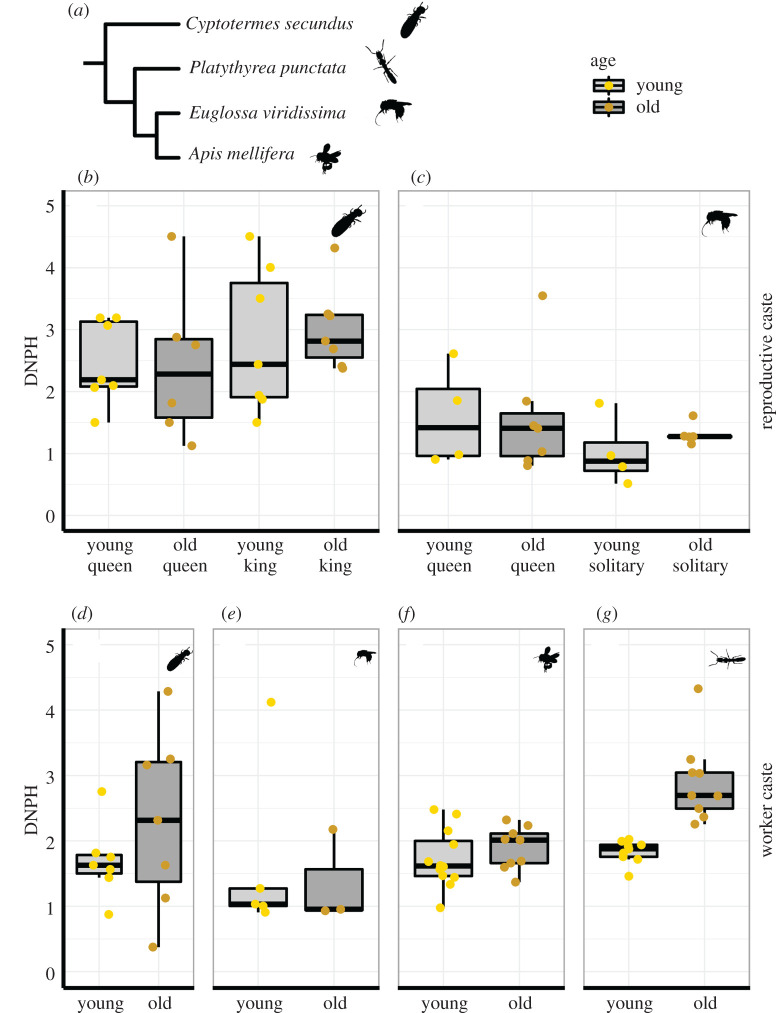
Quantification of carbonyl groups (DNPH assay) in oxidized proteins. (*a*) The phylogeny and the silhouettes of the species tested. (*b*–*g*) The quantification of carbonyl groups (in nmol carbonyl/mg protein) for young and old reproductive individuals of (*b*) the termite *Cryptotermes secundus* (*n* = 7 for each group) and (*c*) the bee *Euglossa viridissima* (solitary young/old: *n* = 4/5, queen young/old: *n* = 4/7) and for young and old non-reproductive individuals of (*d*) *C. secundus* (workers young/old: *n* = 7/7), (*e*) *E. viridissima* (workers young/old: *n* = 5/3), (*f*) the bee *Apis mellifera* (workers young/old: *n* = 12/9) and (*g*) the ant *Platythyrea punctata* (workers young/old: *n* = 9/9). *E. viridissima* is a facultative eusocial bee species; therefore, we distinguished between solitary and social nest structures for reproductive individuals. The boxplots represent the median (bold line), the lower and upper boundaries of the boxes correspond, respectively, to the 25th and 75th percentiles. Each point represents the original datum of one individual. Results of the statistical analysis are shown in table 1 and electronic supplementary material, Methods S1.

Age had no effect on protein oxidation levels in any of the *C. secundus* castes (queens: ER = 1.45, PP = 0.59; kings: ER = 2.3, PP = 0.7; workers: ER = 2.34, PP = 0.7; figure 1*b,d*, table 3, for original sample sizes table 1). Independent of age, oxidation levels were similar in kings and queens (ER = 3.38, PP = 0.77, figure 1*b*) and higher in reproductives of both sexes than in workers (queens versus workers ER = 24.64, PP = 0.9; kings versus workers ER = 118.05, PP = 0.99, table 3).

**Table 3.** Results of linear hypothesis testing within species on the levels of protein oxidation using DNPH. We considered an evidence ratio (ER) of greater than 6 (posterior probability (PP) greater than 0.85) as a trend (indicated in bold font), while we considered an ER of greater than 22 (PP = 0.95) as significant (*) [51]. The table including between-species comparisons can be found in the electronic supplementary material, table S1.

species	caste	age class	versus	caste	age class	evidence ratio	posterior probability
*Apis mellifera*	worker	old	**> **	worker	young	5.38	0.84
*Platythyrea punctata*	worker	old	**> **	worker	young	9999	1.00*
*Euglossa viridissima*	worker	young	**> **	worker	old	1.65	0.62
*Euglossa viridissima*	queen	young	**> **	queen	old	1.21	0.55
*Euglossa viridissima*	solitary	young	**> **	solitary	old	5.29	0.84
*Euglossa viridissima*	queen	all	**> **	worker	all	1.68	0.63
*Euglossa viridissima*	worker	all	**> **	solitary	all	3.18	0.76
*Euglossa viridissima*	queen	all	**> **	solitary	all	6.17	**0.86**
*Euglossa viridissima*	queen	young	**> **	worker	young	1.18	0.54
*Euglossa viridissima*	worker	young	**> **	solitary	young	7.55	**0.88**
*Euglossa viridissima*	queen	young	**> **	solitary	young	8.13	**0.89**
*Euglossa viridissima*	queen	old	**> **	worker	old	1.69	0.63
*Euglossa viridissima*	solitary	old	**> **	worker	old	1.28	0.56
*Euglossa viridissima*	queen	old	**> **	solitary	old	1.35	0.58
*Cryptotermes secundus*	worker	old	**> **	worker	young	2.34	0.70
*Cryptotermes secundus*	queen	old	**> **	queen	young	1.45	0.59
*Cryptotermes secundus*	king	old	**> **	king	young	2.30	0.70
*Cryptotermes secundus*	queen	all	**> **	worker	all	24.64	0.96*
*Cryptotermes secundus*	king	all	**> **	worker	all	118.05	0.99*
*Cryptotermes secundus*	king	all	**> **	queen	all	3.38	0.77
*Cryptotermes secundus*	queen	young	**> **	worker	young	11	**0.92**
*Cryptotermes secundus*	king	young	**> **	worker	young	23.1	0.96*
*Cryptotermes secundus*	king	young	**> **	queen	young	1.83	0.65
*Cryptotermes secundus*	queen	old	**> **	worker	old	6.14	**0.86**
*Cryptotermes secundus*	king	old	**> **	worker	old	23.45	0.96*
*Cryptotermes secundus*	king	old	**> **	queen	old	2.99	0.75

We observed a similar pattern in *E. viridissima*, with no age effects on protein oxidation in queens (ER = 1.21, PP = 0.55, figure 1*c* and table 3), solitary reproductive females (ER = 5.29, PP = 0.84, figure 1*c*) or workers (ER = 1.65, PP = 0.62, figure 1*e*). In this species, there were no obvious differences between the castes, but it seemed that young solitary reproductives had accumulated less oxidative damage to proteins than young individuals living in social nests (young social reproductives versus young solitary reproductives ER = 8.13, PP = 0.89; young workers versus young solitary reproductive ER = 7.55, PP = 0.88, figure 1*c* and table 3).

For *A. mellifera* and *P. punctata*, we only analysed non-reproductive workers. In *A. mellifera* workers, there was no clear effect of age on DNPH (ER = 5.38, PP = 0.84, figure 1*f* and table 3), but old *P. punctata* workers clearly had elevated levels of oxidized proteins when compared to young workers (ER = 9999, PP = 1, figure 1*g* and table 3).

As we sampled thoraces for both bee species, we can directly compare their protein oxidation levels. Workers of *A. mellifera* had a tendency to have more oxidized proteins than workers of *E. viridissima* (ER = 10.33, PP = 0.91, figure 1*e,f*, electronic supplementary material, table S1). This result was driven by old workers of *A. mellifera* having higher levels of DNPH compared to old workers of *E. viridissima* (ER = 8.51, PP = 0.89, figure 1*e*,*f*, electronic supplementary material, table S1). Since we sampled different tissues for the remaining species, we do not compare them here, but the information can be found in the electronic supplementary material, table S1.

Because Vg is a preferred target of carbonylation of the well-characterized *A. mellifera* [6], we checked further with Western blot analysis whether carbonylation was limited to specific proteins or impacted all proteins. Our results showed that several proteins of *E. viridissima*, *A. mellifera*, *C. secundus* and *P. punctata* were carbonylated. We did not find preferentially carbonylated proteins, with the exception of *C. secundus*, where a protein band of approximately 85 kDa shows highest staining intensity (electronic supplementary material, figure S2).

### (b) Antioxidant gene expression

**Figure 2.**
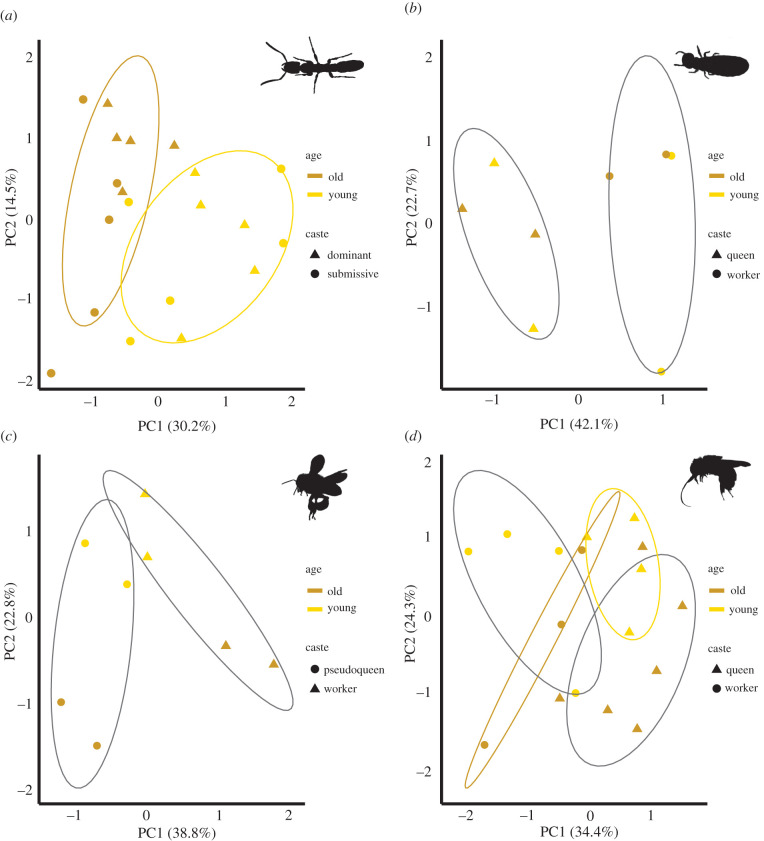
Results of principal component analyses (PCAs) based on the expression of the 20 candidate antioxidant genes common to the four species studied and presented by species: (*a*) the ant *Platythyrea punctata* (abdomen), (*b*) the termite *Cryptotermes secundus* (whole body without guts), and the bees (*c*) *Apis mellifera capensis* (fat body of pseudoqueens and workers and (*d*) *Euglossa viridissima* (abdomen). Shown are the first (abscissa) and second (ordinate) principal components (PC) with variance explained by each PC. Each point represents the expression profile of one individual. Yellow shapes represent young and brown shapes old individuals, triangles represent reproductive individuals and circles, non-reproductive individuals. The ellipses displayed on the PCA plots highlight the main results of the PERMANOVA for each species (see main text), yellow/brown ellipses indicate an age effect, while black ellipses indicate a caste effect.

In *P. punctata* ants, the expression of antioxidant genes changed with age (figure 2*a*; PERMANOVA: *F*_1,19_ = 42.92 *R^2^* = 0.683, *p* < 0.001, table 4), while the reproductive status of an individual did not affect gene expression (*F*_1,19_ = 1.55, *R^2^* = 0.024, *p* = 0.206), nor did the interaction between age and reproductive status (*F*_1,19_ = 2.31, *R^2^* = 0.036, *p* = 0.118). In all other species, reproductive status had a stronger effect than age on antioxidant gene expression (figure 2*b–d*; reproductive status: *C. secundus*: *F*_1,11_ = 3.47, *R^2^* = 0.503, *p* = 0.059 including males, and *F*_1,7_ = 11.93, *R^2^* = 0.690, *p* = 0.024 excluding males; *A. mellifera F*_1,7_ = 7.89, *R^2^* = 0.465, *p* = 0.010; *E. viridissima*: *F*_1,16_ = 9.16, *R^2^* = 0.319, *p* < 0.001). Moreover in *E. viridissima*, age interacted with reproductive status (age: *F*_1,16_ = 3.57, *R^2^* = 0.124, *p* = 0.022, interaction between age and reproductive status: *F*_1,16_ = 3.02, *R^2^* = 0.105, *p* = 0.037), indicating that gene expression changed with age in different ways in reproductives and non-reproductives. In *C. secundus* and *A. mellifera*, we did not find a significant effect of age (age: *C. secundus*: *F*_1,11_ = 0.55, *R^2^* = 0.039, *p* = 0.542 including males, and *F*_1,7_ = 0.84, *R^2^* = 0.049, *p* = 0.419 excluding males; *A. mellifera*: *F*_1,7_ = 3.63, *R^2^* = 0.214, *p* = 0.104, table 4) or significant interaction between age and reproductive status (*C. secundus*: *F*_1,11_ = 0.15, *R^2^* = 0.022, *p* = 0.961 including males, and *F*_1,7_ = 0.52, *R^2^* = 0.030, *p* = 0.547 excluding males; *A. mellifera*: *F*_1,7_ = 1.46, *R^2^* = 0.086, *p* = 0.242).

**Table 4.** PERMANOVA results of the within- and between-species (all species) comparisons. Colons indicate interaction terms. Caste distinguishes between reproductive and non-reproductive individuals.

factor	d.f.	*F*	*R²*	*p*
*E. viridissima*				
age	1	3.567	0.124	0.021
caste	1	9.163	0.318	<0.001
age:caste	1	3.018	0.105	0.037
residuals	13		0.452	
*A. mellifera capensis*				
age	1	3.628	0.213	0.104
caste	1	7.896	0.464	0.010
age:caste	1	1.464	0.086	0.242
residuals	4		0.235	
*P. punctata*				
age	1	42.922	0.683	<0.001
caste	1	1.552	0.024	0.206
age:caste	1	2.310	0.036	0.118
residuals	16		0.254	
*C. secundus* (excl. kings)				
age	1	0.840	0.048	0.419
caste	1	11.933	0.690	0.020
age:caste	1	0.518	0.029	0.547
residuals	4		0.231	
*C. secundus* (incl. kings)				
age	1	0.552	0.039	0.542
caste	2	3.471	0.502	0.059
age:caste	2	0.154	0.022	0.960
residuals	6		0.434	
all species				
species	3	229.530	0.817	<0.001
caste	1	7.592	0.009	<0.001
age	1	18.151	0.021	<0.001
species:caste	3	10.180	0.036	<0.001
species:age	3	9.006	0.032	<0.001
caste:age	1	2.187	0.002	0.010
species:caste:age	3	2.548	0.009	0.019
residuals	61		0.072	

The first PC separated young and old individuals in *P. punctata* (figure 2*a*)*.* It was most strongly influenced by the expression of *Prx3* and *Sod2*, both overexpressed in old relative to young individuals (U-tests: *p* < 0.05, table 2, electronic supplementary material, figure S5C). Old individuals of *E. viridissima* were separated from young individuals by the second PC, which was strongly influenced by a higher expression of *Prx3* in old compared to young individuals (*p* < 0.05, table 2, electronic supplementary material, table S5C). *Prx3* is thus consistently overexpressed in old individuals in both *P. punctata* and *E. viridissima*, which is likely why we also found an age effect in the PERMANOVA results. However, when we specifically investigated the *Prx3* gene in the other two species in which old and young individuals did not differ according to the PERMANOVA results, *Prx3* expression did not differ significantly (*C. secundus* young individuals *x* = 1226 and old *x* = 1185, U-test *p* > 0.99; *A. mellifera* young *x =* 2612, old *x =* 2452, *p* > 0.99).

For the termite *C. secundus*, PC1 separated workers from queens (figure 2*b*). Workers were characterized by relatively high expression of *Cat*, *CG31028, MsrA, Sod1* and *sepia* (table 2, electronic supplementary material, table S5B; U-tests: *p* < 0.05 in all cases), while *GstS3* and *GstZ2* had a relatively high expression in queens (U-tests: *p* < 0.05). In the bee *A. mellifera*, PC1 separated workers from pseudoqueens (figure 2*c*). This PC was characterized by a number of genes with high expression levels in workers (*CG6523, CG8993, GstT3, Mgstl, Prx3, Sod1*; *p* < 0.05 in all cases; table 2, electronic supplementary material, table S5B). In the bee *Euglossa viridissima*, PC1 separated dominant reproductives and workers (figure 2*d*). The three genes contributing most to this PC were *Mgstl*, *Txl* and *Sod1* (table 2, electronic supplementary material, table S5B). All three genes were overexpressed in workers relative to reproductives (U-tests: *p* < 0.01). For the PCs that showed correlation with reproductive status, *Sod1* was consistently overexpressed in workers compared to queens in the three species in that reproductive status affected oxidative gene expression. However, in *P. punctata*, for which we found no caste effect through the PERMANOVA, *Sod1* expression was higher in dominants than in subordinates (subordinate individuals *x* = 1285, dominants *x* = 2690; U-Test *p* < 0.05).

**Figure 3.**
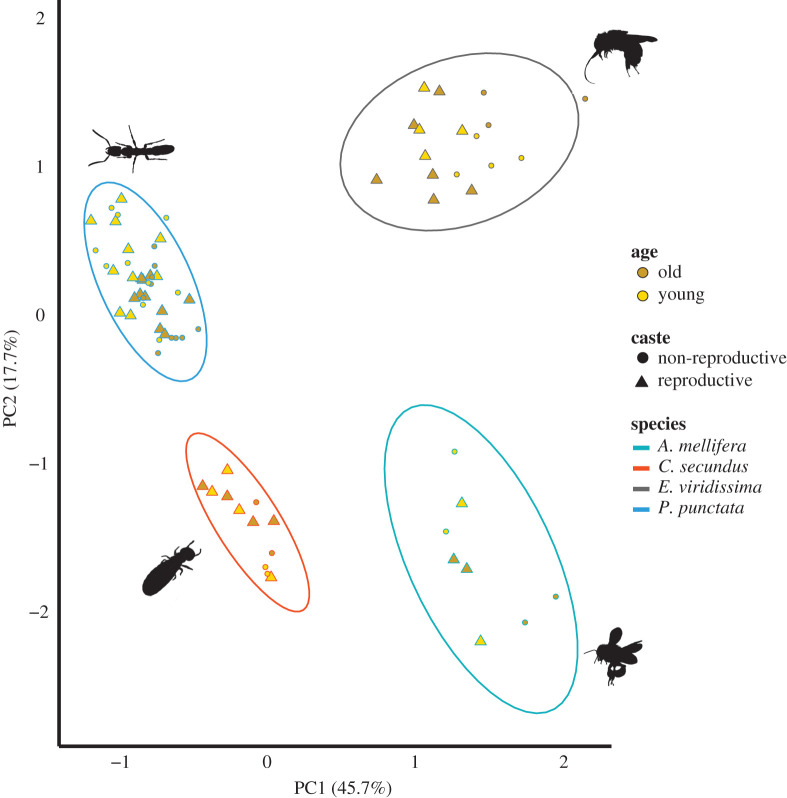
Results of principal component analyses (PCAs) based on the expression of the 20 selected candidate antioxidant genes across four species studied (*Platythyrea punctata*, *Cryptotermes secundus*, *Apis mellifera capensis*, *Euglossa viridissima*). Shown are the first (abscissa) and second (ordinate) principal components (PC) with variance explained by each PC. Each point represents the expression profile of one individual. Yellow shapes represent young and brown shapes old individuals, triangles represent reproductive individuals and circles, non-reproductive individuals. The ellipses (95% confidence interval) displayed on the PCA plots highlight the variable ‘species'. Note that samples of both tissues (head and abdomen) are included for *Platythyrea punctata*.

The between-species PCA revealed that the two PC axes grouped all samples by species (figure 3). The PERMANOVA results showed that the variable ‘species' explained more than 80% (*F*_3,76_ = 229.53, *R^2^* = 0.82, *p* < 0.001) of the variation, while the variables ‘age' or ‘reproductive state' each only explained approximately 3% of the variation (*F*_1,76_ = 18.15/7.59, *R^2^* = 0.02/0.01, *p* < 0.001/<0.001, table 4).

## 4. Discussion

Our results are characterized by a lack of consistency across species, both in terms of protein damage accumulation and antioxidant gene expression. We found a clear increase in carbonyl accumulation with age in the workers of only one out of four species (*P. punctata*) and never within the reproductives. For the two species for which we analysed young and old workers as well as reproductives, we also did not find an age effect. Therefore, the effect of age might be species-specific rather than caste-specific. We found antioxidant gene expression to vary most between species, though a caveat is that we were not always able to analyse the same tissue across the different species. Smaller degrees of variation were found in intraspecific differences between castes or young and old individuals, but not in all species. Overall, the genes differed in expression across species, with the exception of the gene *Sod1*, which was consistently overexpressed in workers relative to queens, and the gene *Prx3*, which was consistently overexpressed in old individuals.

### (a) Inconsistency across species

Because previous work on different species failed to establish a consistent picture of how social insects deal with oxidative stress, we hypothesized that oxidative stress-related responses might vary greatly even between relatively closely related species such as *A. mellifera* and *E. viridissima*. Through our comparative approach, we were able to combine data for protein carbonylation and expression patterns of antioxidant genes across four different species, representing three origins of eusociality [60]. We found variation in the expression of selected antioxidant genes to be much larger between-species than within-species (figure 3). In part, this could have been a consequence of using different tissues in different species (table 1). However, we analysed two different tissues of the same individuals for the ant *P. punctata*. Here, the differences between tissues were much smaller than the differences between the species, indicating that the interspecific variation observed in our study is likely not confounded by tissue-specific expression patterns. Nevertheless, comparisons across species should be interpreted cautiously since variation between samples can be introduced even by small technical differences within the sequencing facilities. Further, we cannot be sure if all genes analysed across the different species are orthologues; this could explain some of the between-species variation.

A similarly inconsistent picture emerged from the analysis of carbonylated proteins: the qualitative and quantitative results varied greatly between species. Workers of the clonal ant *P. punctata* accumulated carbonylated proteins as they aged, but this was less clear for the remaining three species, which additionally did not show an effect of age in the reproductives. Differences between queens and workers were evident only in the termite *C. secundus*, and here reproductives had more carbonylated proteins than workers. For the facultative eusocial bee *E. viridissima*, we only found an interaction between age and caste.

Our results support our initial hypothesis that antioxidative processes vary even across closely related social insect species. This may be one reason why different studies of oxidative stress-related gene expression or oxidative damage often disagree in their findings. These studies are by no means flawed, but one must consider that each species tested might have its own response to oxidative stress. Individuals can prevent oxidative damage in a number of ways (produce only little ROS, neutralize ROS, repair ROS damage or restrict ROS damage to less important tissues), and, depending on the measures taken, we would expect to find more or less protein carbonylation in different tissues. Similarly, genes related to oxidative stress may show different expression patterns between young and old individuals or between castes in one species, but may not give any signal in another species. Without knowledge of the species-specific responses, an interpretation across species may not be meaningful. This should be considered in future studies on oxidative stress.

### (b) Age effects

It is often assumed that older individuals show higher levels of carbonylated proteins than young individuals. Carbonylated proteins are typically degraded by the proteasome and replaced by newly synthesized proteins. When cells do not manage to remove all carbonylated proteins, they can aggregate, are then proteolysis resistant, can inhibit protease functions (reviewed in [11]) and may have negative effects on cellular functioning, thus causing senescence.

Our data of age effects on protein carbonylation of workers in four species clearly indicate carbonyl accumulation in only one of the species (*P. punctata*). In this species, protein carbonyls were measured for the combined tissues of head and thorax. While we also used the thorax in two other species (table 1), we cannot exclude the possibility that differences between species are partly owing to the use of different tissues. *P. punctata* is the only species for which we used the head to measure protein carbonylation, and potentially carbonyl accumulation is strongest in the head. A previous study found no difference between honeybee foragers (old) and nurses (young) when focusing on protein carbonylation in the abdomen [61]. In other honeybee tissues, carbonylation does change with the behavioural transition from hive to outdoor foraging, and though studies disagree on the direction of this effect, carbonylation has been found to be higher in foragers than nurses [26,27]. It is possible that, when different body parts are combined for analysis, the effects on protein carbonylation cancel each other out. In the termite *C. secundus,* old workers appear to have higher carbonylation levels (figure 1*d*) but this result is not statistically supported (table 3), which might be a consequence of high variation in old workers combined with low sample size. In the facultative eusocial bee *E. viridissima*, the average carbonylation levels of old and young workers were virtually identical. This could be owing to the fact that workers of some species do not accumulate damage, perhaps because they deal with oxidative stress as it occurs, e.g. by neutralizing it with enzymatic antioxidants before it can cause damage [14]. However, another reason in the case of *E. viridissima* could be that old workers were not yet in a stage of their life during which they could have accumulated damage. The definition of ‘old' and ‘young' across species is vexed (for details, see electronic supplementary material, Methods S1) because different species have different lifespans and age effects may manifest rapidly only after a threshold age or behavioural transition, especially since age and task are often correlated (e.g. [25]). Furthermore, protein carbonylation is an important irreversible modification in protein quality control and does not *per se* necessarily lead to a damaged cell. It rather acts as a signal to lead the damaged protein to the degradation pathway, which usually effectively removes the protein via proteolytic degradation. Only if carbonylated proteins accumulate or are not efficiently removed do they subsequently aggregate, at which point normal cellular functions are then likely to be disrupted [10,11]. Finally, protein carbonylation is not the only damage that ROS can cause; the damage may be concentrated on other macromolecules like lipids or DNA, or might accumulate in different tissues from the ones we studied, or vary between the sampled species.

Seehuus *et al*. [6] showed that when oxidative stress is induced experimentally, Vg accumulates significantly more carbonyls compared to the two other most abundant haemolymph proteins in honeybees: apolipoprotein and hexamerin. This has been interpreted as preferential oxidation of Vg, substantiating the antioxidant function of Vg [6]. By contrast, we did not find any obvious signs of preferentially carbonylated proteins in *A. mellifera* that might have such an effect in the overall proteome of naturally aged bees (electronic supplementary material, figure S2).

We found a difference in the expression of antioxidant genes with age in only two of our four study species: the facultative eusocial bee *E. viridissima* and the clonal ant *P. punctata*. The reasons why these two species exhibit a change in antioxidant gene expression with age are not clear. While it is possible that individuals of different ages (and hence exhibiting different behavioural repertoires) will experience different types or intensities of oxidative stress, an equally valid assumption would be that ROS are ROS, and whatever machinery is used to deal with them is optimal at any time in the life of an individual. *E. viridissima* and *P. punctata* were also the only two species for which the transcriptomes were generated with the entire abdomen. For the termite *C. secundus*, we used the entire body, and for the Cape honeybee *A. mellifera capensis* only the fat body. While the latter is an important regulatory tissue in the abdomen, there may be other tissues included in the abdomen samples that are responsible for the strong age effects. On the other hand, we also found strong age effects in *P. punctata* heads; it is therefore unlikely that the interspecific differences in age effects we detected are purely caused by differences in the source of tissue used for the different species.

On the level of individual gene expression, there was little consistency across species. Only one gene was strongly associated with age in both species in which old and young individuals clearly differed in their gene expression: *Prx3* was overexpressed in old *P. punctata* ants and *E. viridissima* bees when compared to young individuals. But this was not the case in the other two species, the honeybee and the termite. *Prx3* and *Prx5* double knockdowns experience increased ROS activity and rapidly senescing phenotypes in *Drosophila* [62] because peroxiredoxins maintain the redox state of the cell by eliminating superoxides. Their increased expression with age in *P. punctata* and *E. viridissima* might be related to a dysregulated cellular metabolism in old individuals, with more peroxides being produced by mitochondria, which become leakier with age as a consequence of protein carbonylation. As protein carbonylation increases with age in *P. punctata* but not in *E. viridissima*, the two species may differ in whether increased peroxide levels can be neutralized or where in the body they cause damage.

### (c) The role of reproduction

In the realm of social insects, it is an intuitive assumption that workers take over tasks of colony maintenance, such as feeding the larvae, and may thus accumulate some of the damage that in solitary insects is associated with reproduction, while queens accumulate only little damage as their main task is to produce eggs. That we found carbonyl accumulation with age only in workers (but not across all species), but never in reproductives, is in line with this idea—the presence of workers might reduce oxidative stress in queens. Further support for the idea that a workforce protects reproductives from ageing comes from the observation that only solitary *E. viridissima* reproductives, but not social reproductives, accumulated carbonyls with age. The costs of reproduction might thus, at least in part, occur in the form of oxidative damage and they can be outsourced from reproductives to the workers in social species.

But in contrast with our expectation that workers should show elevated oxidative damage, we found higher carbonylation in *C. secundus* reproductives than workers, a difference that was already present in young individuals. This may be in line with the idea that these workers do not work hard [63] and that workers are totipotent immatures in this species [64]. In fact, a study in which stress was introduced through temperature variation in *C. secundus* also showed that workers mounted their oxidative stress response by increasing the expression of antioxidant genes under stressful temperature conditions more than queens [65]. This was explained by the fact that, as wood-dwelling, one-piece nesters, *C. secundus* workers are totipotent immatures that are selected to invest in body maintenance as they have not reached maturity yet. A previous study on *Reticulitermes speratus* found contrasting results, namely a higher carbonylation rate of worker proteins than queen proteins [16]. The workers of *C. secundus* have a higher totipotency than those of *R. speratus*, in line with different lifestyles of both species (wood-dwelling, one-piece nesting termite versus foraging, multiple-pieces nesting termite) and different development (linear versus bifurcated developmental pathway) [36,66,67]. These differences may explain the contrasting results between both species.

Remarkably, in our study species the different castes—reproductives and non-reproductives—always differed in their expression of genes associated with oxidative stress. Since we analysed these genes in isolation and not relative to genes involved in other pathways, we cannot conclude much about the total investment into defence against ROS, or into the repair of the damage they cause. Still, our results seem to indicate that reproductives and non-reproductives either differ in the oxidative stress they experience, or they employ different mechanisms to deal with this stress. This again highlights the view that reproductive division of labour has strong effects on traits that are closely related to longevity. For *C. secundus*, the results of the temperature stress experiment imply that ROS defence mechanisms are similar across castes but that the castes experience oxidative stress differently [65].

One gene that stood out was *Sod1*, which had a relatively high expression in workers compared to queens in three of our study species, with a reversed expression pattern in *P. punctata*. Superoxide dismutases are known to eliminate harmful reactive oxygen species by catalysing the reaction from highly reactive superoxide into water in both mammals and insects [68]. In *Drosophila*, it has been shown that an overexpression of this gene can prolong a fly's life, and mice *Sod1* knockouts accumulate more oxidative damage in their macromolecules [31]. However, in a previous study on *Lasius niger* ants, the expression of this gene and the activity of its enzyme are actually lower in long-lived queens than in short-lived workers [20]. Our results generalize this finding from the ants to the bees and termites, both of which evolved eusociality and the divergent life histories of queens and workers independently from the ants. At the same time, our previously published results demonstrate that this effect may not necessarily be present in all ant (i.e. *P. punctata*) and termite species (Reticulitermes: [22]), once more highlighting that there are many species-specific idiosyncrasies in oxidative stress management across the social insects.

## 5. Conclusion

Our study may explain why numerous studies on oxidative stress in social insects have failed to give a clear perspective. Most importantly, even after accounting for potential differences in effects owing to the analysis of different tissues, we show that different species show distinct patterns of oxidative stress-related gene expression for the 20 oxidative stress-related genes we investigated in this study. The variation in gene expression among species appears much higher than any variation observed between castes or age classes within a species. The study of carbonyl accumulation gave a similar picture: different species deal differently with oxidative stress and therefore carbonylated proteins can be a predictor of senescence in some species but not others.

## Data accessibility

All data available in the electronic supplementary material.
